# The Paradox of Palliative Care at the End of Life: Higher Rates of Aggressive Interventions in Patients with Pancreatic Cancer

**DOI:** 10.3390/jcm13175286

**Published:** 2024-09-06

**Authors:** Zidong Zhang, Kaushik Gokul, Leslie J. Hinyard, Divya S. Subramaniam

**Affiliations:** Advanced HEAlth Data (AHEAD) Institute & Department of Health and Clinical Outcomes, School of Medicine, Saint Louis University, St. Louis, MO 63104, USA; kaushik.gokul@health.slu.edu (K.G.); leslie.hinyard@health.slu.edu (L.J.H.); divya.subramaniam@health.slu.edu (D.S.S.)

**Keywords:** pancreatic cancer, palliative care, hospital admission, emergency department visit, aggressive intervention, end of life

## Abstract

**Background:** Palliative care has shown benefit in patients with cancer; however, little is known about the overall utilization of palliative care services in patients with pancreatic cancer and the impact of aggressive end-of-life interventions. This study aimed to explore the incidence of palliative care consultations (PCCs) in hospitalized patients with pancreatic cancer in the United States and the association between palliative care consultations and the use of aggressive interventions at the end of life. **Methods:** We conducted a retrospective study of patients hospitalized with pancreatic cancer. We examined patient records for 6 months prior to death for the presence of PCCs and aggressive end-of-life (EOL) interventions—emergency department visits, chemotherapy, and ICU stays. The use of EOL interventions was compared between those who did and those who did not receive PCCs, using Chi-square and Whitney U tests. **Results:** Of the 2883 identified patients, 858 had evidence of a PCC in their record in the last 6 months of life. Patients receiving PCCs were older at the time of death and more likely to receive chemotherapy (22.4% vs. 10.6%) in the last 6 months of life compared to those not receiving a palliative care consult. Similarly, patients with PCCs were more likely to have aggressive interventions in the EOL period. **Conclusions:** Less than 30% of patients with pancreatic cancer received a PCC. Those who received a PCC had more aggressive interventions in the end-of-life period, differing from what the prior literature has shown. Future investigations are necessary to explore the components and timing of PC and investigate their influence on the utilization of aggressive interventions and patient-centered outcomes.

## 1. Introduction

Pancreatic cancer, specifically cancer of the exocrine pancreas, is the fourth leading cause of adult mortality in the United States, regardless of gender and race. In 2024, it is estimated that there will be 66,440 new cases and 51,750 in the US [1]. The 5-year survival rate of pancreatic cancer is around 10%, notably lower in comparison to other types of malignancies [2]. This low survival rate is predominantly because symptoms usually present late in the disease course, and consequently, the cancer has advanced to local and regionally advanced stages at the time of diagnosis. Only approximately 10% of pancreatic neoplasms are identified as localized, thus allowing for surgical resection [3].

In current practice, surgical treatment is the only curative treatment option available, whereas other treatment options for pancreatic cancer, such as radiation, chemotherapy, and combinations of these approaches, can convert some advanced cases to be surgically resectable and extend survival [4,5]. Strides have been made in terms of the advancement of treatment options with refined surgical techniques and advanced medication regimens such as FOLFIRINOX, currently being employed with much improved survival and efficacy compared to prior treatments [6]. However, late presentation of pancreatic cancer often necessitates aggressive interventions, resulting in a higher financial burden with limited ability to prolong life [7].

Additionally, the mortality rate of pancreatic cancer remains high, primarily due to the late stage at diagnosis, despite advancements in treatment approaches [2,3]. Thus, prioritizing patient comfort during aggressive treatment with curative intent and during end-of-life (EOL) intervention is desirable.

Palliative care (PC) is an interdisciplinary treatment approach aimed at enhancing quality of life for patients living with a chronic life-threatening illness, often administered to patients in advanced stages [8,9]. In contrast to hospice care, PC emphasizes optimized patient-centered outcomes, including the adjustment of treatment plans for the patient’s comfort and wishes for care, the management of mental health issues related to the diagnosis of deadly illnesses, and the reduction in unnecessary treatment based on the patient’s needs [9,10,11,12,13]. Hereby, PC can reduce hospital admissions, emergency department (ED) visits, and costs for hospital-related services in patients with serious and complicated illnesses [14,15,16,17]. Considering the significant financial burden of cancer treatment, palliative care may help improve survival and adherence to treatment due to cost [18,19]. However, such improvement in health economics and the value of health services can vary by the context of care; for example, many findings are from clinical trials. Still, little is known about the uptake of PC and the influence on the medical interventions in the EOL of patients with pancreatic cancer amid the complexity of treatment and the heavy symptom burden. Among the nuances of palliative care, such as the referral process, content, and delivery of PC, it is fundamental to determine the utilization of PC consultations in a national patient sample and to study the impact on the use of aggressive interventions, such as chemotherapy, hospital admissions, ED visits, and Intensive Care Unit (ICU) stays.

## 2. Study Purpose

This study aims to examine the demographics of patients with pancreatic cancer who received palliative care referrals in the last 6 months and the last 30 days of life. A secondary aim is to compare the utilization of aggressive interventions near the end of life between patients with pancreatic cancer who received a palliative care referral and those who did not.

## 3. Methods

### 3.1. Data Source

This study utilized the Optum^®^ de-identified Integrated Claims-Clinical dataset (the integrated dataset) between 1 January 2000 and 31 December 2018 (Optum Inc., Eden Prairie, MN, USA). The data were derived from health systems in the United States, including more than 740 hospitals and 7000 clinics treating more than 104 million patients receiving care in the United States. Available data included outpatient and inpatient medical services, diagnostic codes, procedure codes, laboratory values, and prescribed and administered medications. Encounter settings included academic, non-academic, and safety net healthcare facilities. Source data were a sample of 5 million non-institutionalized adult patients (≥18 years) throughout the United States who were commercially insured, government insured, and uninsured. The current study was deemed exempt by the Saint Louis University Institutional Review Board.

### 3.2. Study Population

This retrospective cohort study included all deceased patients in the source dataset with a diagnosis of pancreatic cancer (ICD-9-CM 157.1–157.9, 211.6–211.7, V10.09 and ICD-10-CM C25-C25.9, D13.7-D13.7, Z85.07). The codes were all-encompassing and included diagnoses of both malignant and benign pancreatic cancers. The primary predictor variable of interest was the presence of a palliative care consultation (PCC) in the 6 months prior to death. A PCC was identified with an ICD-9 V66.7 or ICD-10 Z51.5 code, and patients with an ICD-9/10 code for a palliative care consultation within the last 6 months of life were classified as receiving a PCC. These codes have good sensitivity, ranging from 53.9% to 84%, and high specificity (over 90%) [20,21,22]. Many published studies have used these codes to examine administrative databases for palliative care consultations and encounters [17,23,24,25,26,27]. Aggressive interventions at the EOL were captured using ICD-9/10 codes (Supplementary Table S1). Aggressive interventions were defined in this study as any number/amount of chemotherapy, ICU stays, hospitalizations, or emergency department visits within the last 6 months and the last 30 days of life. We used this period because it is commonly used for investigations of palliative care utilization [23,28]. Demographic factors of interest included age at the time of death, gender, race, household income, and geographic region. The demographic and sociodemographic variables were extracted from the patient file datasets associated with the integrated dataset. Age of death was derived from the reported birth and death years. Gender, race, and ethnicity were self-reported. The socioeconomic variables (region, average household income per ZIP, and proportion of individuals with college education per ZIP) were available in the source dataset. There were no uninsured patients in the sample.

### 3.3. Statistical Analysis

Continuous variables, including mean and standard deviation, were compared between patients having a PCC and those without a PCC at the end of life using Student’s *t*-test. The categorical variables were compared in proportions between patients using Chi-square tests. The correlations between health service utilizations in the last 6 months and the last 30 days of life were assessed using McNair’s test. All of the tests were two-tailed, with an alpha equal to 0.05. The statistical analysis was conducted with SAS v9.4 (SAS Institute, Cary, NC, USA).

## 4. Results

### 4.1. Patient Characteristics

A total of 2883 patients with a diagnosis of pancreatic cancer meeting our inclusion criteria were identified in the database, of whom 858 (29.8%) received a PCC in the last 6 months of life. Table 1 presents the patient characteristics stratified by PCC. Patients who received a PCC were older at the time of death than those who did not receive a PCC (70.5 years vs. 68.9 years, respectively; *p* < 0.001). There was a slightly higher proportion of men in the non-PCC group compared to the PCC group (54.5% vs. 50.5%, *p* = 0.05) and a greater proportion of Black patients in the PCC group (*p* < 0.001). There were also geographic differences in terms of receipt of a PCC, with a higher proportion of patients receiving palliative care in the Midwest (*p* < 0.001). There were no statistically significant differences in household income and the level of education between the two groups.

### 4.2. Utilization of Aggressive Interventions in the End of Life

The comparisons of late-stage interventions between the PCC and non-PCC groups within the last six months of life are shown in Table 2. All categories of aggressive intervention—chemotherapy, ICU stays, and ED visits—were higher in patients who received a PCC compared to those who did not. In total, 22% of patients with a PC consult received chemotherapy in the last 6 months of life compared to 10.6% of those without a consultation (*p* < 0.001). Similar patterns were seen for hospitalizations (81.1% vs. 38.2%), ED visits (45.1% vs. 23.7%), and ICU stays (14.1% vs. 4.0%) for patients with a PC consult compared to those without a consult. The average number of ED visits, number of hospital admissions, length of hospitalization, and length of ICU stays were also higher for patients who received a PC consult compared to those who did not (*p* < 0.001, Table 2). Similar patterns were observed when examining interventions in the last 30 days of life (Table 3).

## 5. Discussion

Pancreatic cancer accounts for approximately 3% of cancers but makes up 7% of cancer-related deaths in the US [3]. To date, there has been limited research examining the impact of palliative care on patients with pancreatic cancer specifically. Since pancreatic cancer is usually advanced at the time of diagnosis, these patients are more likely to advance fast and have shorter survivorship compared to those with other common cancers like breast cancer and lung cancer [29]. Therefore, patients with pancreatic cancer should have urgent needs for PC consultation following cancer diagnosis. In our study, we reported that in the last 6 months of life, less than a third of patients with pancreatic cancer received a PCC, and the patients who received a PCC had greater proportions of ED visits, ICU stays, and chemotherapy administration than those who did not. This study will help better inform providers about the impacts of palliative care on patients with pancreatic cancer.

There were limited studies on the effect of palliative care on the utilization of aggressive interventions at the end of life for patients with advanced cancer. A Canadian study reported that a PCC appointment was associated with a reduced number of aggressive treatment indicators toward the EOL in non-resectable pancreatic cancers, including ED visits, chemotherapy, and ICD admissions [30]. Unlike these studies, Bhulani et al. found that in a SEER-Medicare cohort, patients with pancreatic cancer who received a PCC had a significantly greater number of average ED visits within the last 30 days (about 4.5 weeks) of life and higher costs of care compared to patients who did not [23]. Moreover, in this same study, 70% of patients who received their first PCC had received it within the last 30 days of life, and nearly one-third (31.6%) of patients received their first PCC within the last 7 days of life [23]. Likewise, Zhang et al. found a high correlation between receipt of a PC consultation in the EOL period and in the initial 2 months of metastatic breast cancer, and that the patients who received PC in the earlier days of metastatic breast cancer were still more likely to use aggressive interventions. This indicated that PC in the EOL period may not cause the higher utilization of aggressive intervention in the EOL period for patients with aggressive cancer, but instead, it indicates the higher symptom burden of those patients who have received palliative care, even starting from when metastatic cancer was diagnosed; the symptom burden of these patients has created the need for both palliative care and aggressive interventions [31]. We understand that PCCs might reduce the utilization of aggressive interventions in some patient groups, but those pertinent studies could be subject to selection bias from their populations, such as subjects in clinical trials or Medicare beneficiaries with exclusions to certain medical complexity. Additionally, the variation in the effect of PCCs on the use of aggressive interventions can also be attributed to the different timeframe of the defined EOL period per se. Because the intensity of disease-modifying treatment wanes when a patient approaches death, it is not surprising that the utilization of aggressive intervention can be higher in a longer EOL period, such as the last 30 days of life, compared to a shorter EOL period like the last 14 days of life. The medical interventions in the last 14 days of life could be adjusted to align with the end-of-life conversation, which prescribed the termination of many aggressive interventions [28]. This could contribute to the unmeasured misclassification, due to the unknown transition from palliative therapy (concomitant to the standard, disease-modifying therapy) to hospice care in a shorter timeframe of EOL. However, both measurements have been used in published studies. Several articles reported that patients with PC had a higher number of ED visits, unplanned hospital admissions, ICU stays, and overall aggressive interventions in the last 30 to 90 days before death, rather than the last 14 days; these include several articles covering the ENABLE II trial [32], a cross-sectional hospitalized sample of elderly patients with pancreatic cancer [23], and cohorts of breast and gastric cancer [33,34].

Conflicting findings like those by Bhulani et al. [23] and those by Bevin et al. [17] might portray the actual health service pattern amid the complexity of care for patients with advanced illness, especially care at the end of life. It is not rare for patients with advanced cancer to visit the ED for symptom management, especially those patients who are in hospice care, regardless of location, with symptomology beyond the capability of their primary medical provider [35,36]. Treatment for addressing other aspects of advanced cancer can also coincide with PC in the EOL period but prior to hospice care [31]. Moreover, some patients are dying in the ICU, so their acuteness of condition justifies referrals to PC [23]. The literature implies that the relationship between PCCs and the utilization of hospital and ICU resources can vary in the real world. Therefore, the aggressiveness of pancreatic cancer could explain the co-existence of the higher utilization of aggressive interventions and PC administration at the end of life, as was observed in real-world data. Thus, we believe the higher utilization of aggressive intervention in the EOL period can be explained by the need for specialty symptom management. Palliative care should be integrated into the standard care for pancreatic cancer as early as possible, as the early integration may help plan for and create timely access to symptom management and other palliative treatments in order to prepare for cancer advancement and, in the meantime, reduce avoidable hospital and ED visits before the EOL. Bevin et al.’s study supports this recommendation [17]. Yet, the patient’s comfort and symptom burden are not available in most administrative databases [9].

Our study might be affected by a few limitations. Like any investigations using administrative databases, some patients in our cohort may have received a consultation appointment and then not received additional services that could be classified under the umbrella of palliative care. On the other hand, some patients may have received multiple palliative care treatments following a PCC without the time and content of treatments documented in the EHR. Thus, it is not possible to accurately determine the exact start and end dates of PC and the time span of PC services. Likewise, it may be even less accurate to determine the use of palliative care in the shorter period at the EOL, for instance, the last few days of life, because of the possible undocumented PC treatments. Second, the association between PC uptake and healthcare utilization does not mean causality. While some clinical trials reported reduced utilization of admissions to hospitals, EDs, or ICUs, the associations can be demonstrated differently in patients’ records, as we found. Although observational studies can barely determine causal relationships, these investigations may unveil components in health services where improvements can be made. Third, the coding within administrative databases does not provide nuanced information of palliative care consultations and palliative care services. This means the code for PCCs may encompass primary and specialist palliative care, including consultations and specific treatments. Thus, the uptake of PCCs might be underestimated in some settings. However, we did not think this would affect our result much because the aggressive nature of pancreatic cancer and the complexity of advanced cancer would justify PCCs rather than some primary PC treatments conducted without prior physician-participating consultations. In addition, our analysis included considerable periods (0–6 months) for identifying the presence of PCCs. Since specific periods were not set as parameters in our study (early vs. late), it is difficult to identify when the PCC itself was provided in a patient’s clinical course. This may have impacted the results observed. On the other hand, the 6-month period offers a context closer to real-world healthcare, rather than a group of patients in the EOL period for clinical trials, and thus may make our findings more instrumental to clinical practice. Last, the sample analyzed a higher proportion of patients residing in the Midwest (45.7%), making it biased in terms of geographic representation.

This study has several notable strengths. First, this study was built on a national all-payer EHR database, which enabled the sampling of patients who were commercially insured and those insured by Medicare across distinct parts of the United States. Most studies on the US national PC use among cancer patients employ the SEER-Medicare database, which excludes younger and healthier populations. The nationally distributed sample that includes commercially insured populations in this study improves the generalizability of our results. Second, we implemented a previously validated approach to identify PCCs in the administrative data. In an electronic health record review of data from the Veterans Aging Cohort Study, the reported sensitivity of the ICD-9 code (V66.7) used for indicating PCCs and PC services was 84% and the specificity was 98% [22]. Although we might miss some PC treatments independent from a PCC, we are confident that most cases were captured. Last, our investigation in the last 6 months of life is longer than many existing investigations of PC for EOL, and thus may bring better practical meaning to the outcome evaluation of service utilization.

## 6. Conclusions

Based on our results, palliative care consultations are underutilized in patients with pancreatic cancer within the United States, even in the last 6 months of life. Patients who received PCCs in the EOL period utilized more aggressive interventions, but this can be explained by the need to address the symptom burden of advanced cancer. To create timely access to symptom management and other PC services, effort should be made to integrate PC in the earlier days following cancer diagnosis and to increase PC uptake for all patients with pancreatic cancer. Further study into the decision making of providers who treat patients with pancreatic cancer may shed light on why this is the case. In addition, future research is necessary to explore the components and timing of palliative care concomitant to the standard care for cancer and how they impact patient outcomes and the utilization of aggressive interventions following cancer diagnosis and in the EOL period of patients with pancreatic cancer. The findings from these investigations will help us better understand care delivery and optimize personalized care.

## Figures and Tables

**Table 1:** Demographic information.

	Overall	No PC in Last 6 Months	PC in Last 6 Months	
	N = 2883	N = 2025	N = 858	
Item	Mean (Std Dev)	Mean (Std Dev)	Mean (Std Dev)	*p* Value
Age of death, year	70.0 (11.1)	68.9 (11.8)	70.5 (10.8)	<0.01
Average household income, USD	42,366.8 (10,610.5)	42,508.7 (9601.8)	42,306.7 (11,011.7)	0.63
Percent of college education in community	23.8 (7.6)	23.8 (7.1)	23.8 (7.8)	0.98
	Median (IQR)	Median (IQR)	Median (IQR)	*p* value
Age of death, year	71 (62–79)	72 (63–79)	69.5 (60–79)	<0.01
Average household income, USD	39,689.5 (35,229–47,491)	39,636 (35,113–46,866)	39,766 (35,814–49,165)	0.10
Percent of college education in community	23 (18–27)	22 (18–27)	23 (18–27)	0.47
	%	%	%	*p* value
Gender				0.05
Female	46.7	45.5	49.5	
Male	53.3	54.5	50.5	
Race/Ethnicity				<0.01
Non-Hispanic Caucasian	78.2	78.2	78.3	
Non-Hispanic African	9.0	7.5	12.6	
Hispanic	2.3	2.1	2.8	
Other/Unknown	10.5	12.3	6.3	
Region				<0.01
Midwest	45.7	43.0	52.1	
Northeast	16.7	17.5	14.8	
Other/Unknown	1.8	1.8	1.9	
South	27.0	28.6	23.2	
West	8.7	9.0	8.0	
Division				<0.01
East North Central	29.1	26.9	34.3	
East South Central	4.6	4.7	4.3	
Middle Atlantic	8.1	8.6	6.8	
Mountain	5.8	6.2	4.9	
New England	8.6	8.9	8.0	
Other/Unknown	1.9	1.9	1.9	
Pacific	2.9	2.8	3.2	
South Atl/West South Crl	22.4	24.0	18.9	
West North Central	16.6	16.1	17.8	
Average household income per ZIP				0.11
≤35,229	24.5	25.2	22.8	
>35,229 to 39,689	26.7	26.5	27.4	
>39,689 to 474,913	24.4	25.0	22.8	
≥474,913	24.4	23.3	26.9	
Education				0.01
≤18%	26.9	28.0	24.5	
>18% to 23%	28.0	27.2	29.8	
>23% to 27%	22.2	21.0	24.9	
≥27%	22.9	23.8	20.8	

**Table 2:** Outcomes over last six months of life.

	Overall	No PC in the Last 6 Months	PC in the Last 6 Months	
	N = 2883	N = 2025	N = 858	
	%	%	%	*p* value
ACP ordered	4.5	1.6	11.2	<0.01
Any chemotherapy	14.1	10.6	22.4	<0.01
Any hospitalization	51.0	38.2	81.1	<0.01
Any ED visit	30.0	23.7	45.1	<0.01
Any ICU stay	7.0	4.0	14.1	<0.01
	Mean (Std Dev)	Mean (Std Dev)	Mean (Std Dev)	*p* value
Number of ED visits	0.6 (1.1)	0.4 (1.0)	0.9 (1.4)	<0.01
Number of hospitalization admissions	1.0 (1.3)	0.7 (1.1)	1.6 (1.5)	<0.01
Total number of days of hospitalization	6.5 (15.9)	4.4 (14.3)	11.4 (18.4)	<0.01
Total number of days staying in ICU	0.3 (2.1)	0.2 (1.2)	0.7 (3.4)	<0.01
	Median (IQR)	Median (IQR)	Median (IQR)	*p* value
Number of ED visits	0 (0–1)	0 (0–0)	0 (0–1)	<0.01
Number of hospitalization admissions	1 (0–1)	0 (0–1)	1 (1–2)	<0.01
Total number of days of hospitalization	1 (0–8)	0 (0–5)	7 (2–14)	<0.01
Total number of days staying in ICU	0 (0–0)	0 (0–0)	0 (0–0)	<0.01

**Table 3:** Outcomes over last 30 days of life.

	Overall	No PC in the Last 6 Months	PC in the Last 6 Months	
	N = 2883	N = 2025	N = 858	
	%	%	%	*p* value
ACP ordered	1.7	0.4	4.8	<0.01
Any chemotherapy	1.9	1.3	3.4	<0.01
Any hospitalization	26.1	15.5	51.3	<0.01
Any ED visit	7.8	4.8	14.8	<0.01
Any ICU stay	3.4	1.5	7.9	<0.01
	Mean (Std Dev)	Mean (Std Dev)	Mean (Std Dev)	*p* value
Number of ED visits	0.1 (0.4)	0.1 (0.3)	0.2 (0.4)	<0.01
Number of hospitalization admissions	0.3 (0.5)	0.2 (0.4)	0.6 (0.6)	<0.01
Total number of days of hospitalization	2.5 (11.3)	1.7 (12.5)	4.4 (7.3)	<0.01
Total number of days staying in ICU	0.1 (0.9)	0.1 (0.7)	0.3 (1.3)	<0.01
	Median (IQR)	Median (IQR)	Median (IQR)	*p* value
Number of ED visits	0 (0–0)	0 (0–0)	0 (0–0)	<0.01
Number of hospitalization admissions	0 (0–1)	0 (0–0)	1 (0–1)	<0.01
Total number of days of hospitalization	0 (0–1)	0 (0–0)	1 (0–7)	<0.01
Total number of days staying in ICU	0 (0–0)	0 (0–0)	0 (0–0)	<0.01

## Data Availability

The datasets presented in this article are not readily available because it is a sample of a proprietary EHR database that we leased. Requests to access the datasets should be directed to Optum, Inc.

## Supplementary Materials

The following supporting information can be downloaded at: https://www.mdpi.com/article/10.3390/jcm13175286/s1, Table S1: Aggressive Intervention Codes.
