# Supplementary material for: The Paradox of Palliative Care at the End of Life: Higher Rates of Aggressive Interventions in Patients with Pancreatic Cancer
Source: J Clin Med. 2024 Sep 6;13(17):5286. doi: 10.3390/jcm13175286 (PMC11395880; doi:10.3390/jcm13175286)
Supplement: Supplementary file 1 [file jcm-13-05286-s001.zip › jcm-3121560-supplementary.pdf]

Supplementary Table S1. Aggressive Intervention Codes

Diagnosis/Treatment	Code Type	Code
**Intensive Care**	Revenue code	0200 - 0204, 0207 - 0212, 0219
**Emergency Department Visit**	CPT4	99281 - 99285
	HCPCS	G0380 - G0384
	Revenue Code	0450 - 0459, 0981
**General Chemotherapy**	HCPCS	J9999
**Gemcitabine (Gemzar)**	HCPCS	J9198
**5-fluorouracil (5-FU)**	HCPCS	J9190
**Irinotecan (Camptosar)**	HCPCS	C9474
**Oxaliplatin (Eloxatin)**	HCPCS	J9263
**Albumin-bound paclitaxel (Abraxane)**	HCPCS	J9264
**Capecitabine (Xeloda)**	HCPCS	J8520, J8521
**Cisplatin**	HCPCS	J9060, J9062
**Paclitaxel (Taxol)**	HCPCS	J9264, J9267
**Docetaxel (Taxotere)**	HCPCS	J9171
**Irinotecan liposome (Onivyde)**	HCPCS	C9474, J9206
**Leucovorin**	HCPCS	J0640
